# Study on decision-making for orthodontic treatment plans based on analytic hierarchy process

**DOI:** 10.1186/s12903-024-04281-y

**Published:** 2024-04-24

**Authors:** Chenglu Ruan, Jianying Xiong, Zhihe Li, Yirong Zhu, Qiongqiong Cai

**Affiliations:** Department of stomatology, Sanming Integrated Medicine Hospital, Sanming, Fujian China

**Keywords:** Orthodontics, Analytic hierarchy, Treatment plan, Semantic analysis

## Abstract

**Background:**

Orthodontics is a common treatment for malocclusion and is essential for improving the oral health and aesthetics of patients. Currently, patients often rely on the clinical expertise and professional knowledge of doctors to select orthodontic programs. However, they lack their own objective and systematic evaluation methods to quantitatively compare different programs. Therefore, there is a need for a more comprehensive and quantitative approach to selecting orthodontic treatment plans, aiming to enhance their scientific validity and effectiveness.

**Methods:**

In this study, a combination of the analytic hierarchy process (AHP) and semantic analysis was used to evaluate and compare different orthodontic treatment options. An AHP model and evaluation matrix were established through thorough research and semantic analysis of patient requirements. This model considered various treatment factors. Expert panels were invited to rate these factors using a 1–9 scale. The optimal solution was determined by ranking and comparing different orthodontic treatment plans using the geometric mean method to calculate the weights of each criterion.

**Results:**

The research indicates a higher preference for invisible correction compared to other orthodontic solutions, with a weight score that is 0.3923 higher. Factors such as comfort and difficulty of cleaning have been given significant attention.

**Conclusion:**

The Analytic Hierarchy Process (AHP) method can be utilized to effectively develop orthodontic treatment plans, making the treatment process more objective, scientific, and personalized. The design of this study offers strong decision support for orthodontic treatment, potentially improving orthodontic treatment outcomes in clinical practice and ultimately enhancing oral health and patients’ quality of life.

## Introduction

Orthodontics is a crucial field within dentistry that focuses on the study and treatment of abnormal tooth and jaw alignment, as well as bite problems [1]. Its primary objective is to improve the patient’s oral function and appearance by preventing, diagnosing, and treating dental and maxillofacial misalignments and asymmetries. Achieving good occlusal function and an aesthetically pleasing facial appearance are the core goals of orthodontics [2]. Incorrect tooth alignment and bite problems can have a negative impact not only on an individual’s oral health but also on their self-confidence and mental well-being [3]. Orthodontists utilize their knowledge of teeth, jaws, and the face to design and implement various orthodontic treatment methods, ensuring that patients achieve healthy and comfortable occlusal function while obtaining satisfactory aesthetic results [4]. These treatment methods include traditional fixed braces, removable braces, and invisible braces, among others [5]. Various orthodontic treatment methods, such as traditional fixed braces, removable braces, and invisible braces, are employed to correct malocclusions and improve oral health and aesthetics. Due to variations in patients’ oral conditions and needs, selecting the most suitable orthodontic solution has become a complex and critical issue [6]. Currently, many patients rely mainly on the doctor’s clinical experience and professional knowledge when choosing orthodontic plans, lacking their own objective and systematic evaluation methods to quantitatively compare different plans. This leads to inconsistencies in the effectiveness and satisfaction of the treatment process. Semantic analysis methods are a category of computer science techniques that focus on understanding and interpreting the meaning of human language [7]. Their main objective is to enable computers to gain a deeper understanding of the context, grammatical structure, and semantic relationships within text. This, in turn, facilitates more advanced text comprehension tasks. These methods involve the use of natural language processing techniques to extract information from large-scale textual data, identify associations between words, phrases, and sentences, and capture the meaning conveyed by language expressions more accurately [8].

In the field of semantic analysis, word vector models play a crucial role by mapping words to a vector space. This vector space allows for semantically related words to be closer in distance [9]. By calculating distances between vectors, computers can determine semantic relationships between words. Moreover, techniques like syntactic parsing and semantic role labeling assist in identifying various constituents within sentences and their relationships, thereby improving the understanding of sentence structure [10].

Sentiment analysis is a significant application of semantic analysis methods that aim to determine the emotions or sentiments expressed within text [11]. Through the analysis of vocabulary, context, and grammatical structure, computers can automatically discern whether the text is positive, negative, or neutral. Another application domain is text classification, where training classification models categorize text into different classes. By combining natural language with computer science, semantic analysis methods enable computers to gain a deeper understanding of the meaning of human language. As a result, they play a vital role in fields such as text comprehension, information extraction, and sentiment analysis.

The Analytic Hierarchy Process (AHP) is a multi-criteria decision analysis method used for complex decision-making and evaluations. This method was introduced by American operations researcher Thomas L. Saaty in the 1970s and has been widely applied in fields such as management, engineering, economics, and others [12–14]. AHP involves breaking down complex decision problems hierarchically into interconnected sub-problems, making decision-making more manageable [15]. These sub-problems are structured into a hierarchy based on their importance in the overall decision, which includes the goal level, criteria level, and alternative level. Within each level, decision-makers use pairwise comparison matrices to assess the relative importance of different factors [16].

The Analytic Hierarchy Process (AHP) involves constructing judgment matrices and utilizing pairwise comparisons to assess the relative importance of different factors [17]. This process assists decision-makers in making rational decisions by calculating weights for each factor and determining the optimal alternative. The AHP method offers several advantages in handling multi-criteria decision problems. It effectively considers the relative importance of different factors, which helps to reduce subjectivity and arbitrariness [18]. The Analytic Hierarchy Process (AHP) begins by placing the decision problem within a larger system that consists of multiple interconnected factors [19]. To organize these issues in a hierarchical manner, a multi-level analytical structure model is created. Then, a combination of mathematical methods and qualitative analysis is used. By conducting a hierarchical ranking process, the final decision is made by assigning weights to each alternative based on calculations [18, 20].

In the healthcare field, there is a growing focus on important topics such as performance measurement, risk management, and decision-making. Numerous studies have extensively explored these topics using multi-criteria decision-making methods like the Analytical Hierarchy Process (AHP) and other techniques. For instance, some scholars have utilized the AHP method to predict the priority of ICU admission for critically ill patients, aiming to assist medical institutions in making informed decisions [21]. Furthermore, by employing methods like HFACS, fuzzy TOPSIS, and AHP, researchers have successfully identified significant human error factors in the emergency department [22]. Additionally, there have been studies on medical service performance, medical strategies, and the design of home medical products for the elderly, all of which employ varying degrees of AHP and other methods to provide support for medical decision-making [23–26]. These studies highlight the extensive application of multi-criteria decision-making methods in the medical field, offering powerful tools and techniques to enhance the quality and efficiency of medical care.

The purpose of this study is to develop an orthodontic program evaluation model using AHP and assess its feasibility and effectiveness in clinical practice. Our goal is to investigate the correlation between patients’ oral conditions and treatment needs, and employ semantic analysis methods to identify key indicators. This will facilitate the establishment of an objective, scientific, and quantitative evaluation model. By conducting a comprehensive evaluation of orthodontic treatment options, we aim to provide clinicians with a decision-making framework and offer patients more personalized treatment choices. Additionally, we explore the application of the AHP method in orthodontics and its empirical research value in oral medicine. This research endeavors to advance orthodontic treatment, enhance treatment outcomes and patient satisfaction, and contribute to the enhancement of oral health and aesthetics.

## Method and materials

### Ethics

The study protocol was approved by the research ethics board at Sanming Integrated Medicine Hospital (approval No. 2023-KY-010), and written informed consent was obtained from all patients before the study commenced. The study was conducted in accordance with the revised principles of the Helsinki Declaration [27]. All methods in this study were performed in compliance with relevant guidelines and regulations.

### Research design

This study utilizes a combination of semantic analysis method and the analytic hierarchy process (AHP) to conduct a quantitative analysis of orthodontic solutions. The research design involves the following key steps:

1. Application of semantic analysis method: This stage involves an in-depth study of patient needs and market status in the field of orthodontics through text mining, keyword extraction, and topic analysis. By processing and organizing large amounts of text data, the study identifies key factors and main concerns affecting patient selection, providing a basic framework for subsequent research.
2. Application of AHP method: The study uses the Analytical Hierarchy Process (AHP) to establish an evaluation model for orthodontic programs. This model hierarchizes treatment factors such as comfort, treatment effect, and concealment, and develops a corresponding scoring system. The weight allocation process involves the participation of an expert group who scores each indicator using the 1–9 scale method. This quantitative analysis allows the study to objectively determine the relative importance of each element in an orthodontic program, providing an objective basis for program selection.
3. Data integration and analysis: The study integrates and comprehensively analyzes the results of semantic analysis and AHP evaluation. By comparing and weighing qualitative and quantitative data, the study aims to extract the most constructive opinions and suggestions for orthodontic solution selection.

### AHP hierarchical analysis calculation steps

The steps to determine weights using the Analytic Hierarchy Process (AHP) are as follows:

1. To construct the judgment matrix, we assign A as the objective and u_i_ and u_j_ (i, j = 1, 2, ⋯, n) as the factors. u_ij_ represents the numerical measure of the relative importance of u_i_ to u_j_. These u_ij_ values are then used to form the A - U judgment matrix P.

$$P = \left[{\begin{array}{*{20}{c}}{{u_{11}}}&{{u_{12}}}& \ldots &{{u_{1n}}} \\ {{u_{21}}}&{{u_{22}}}& \ldots &{{u_{2n}}} \\ \vdots & \vdots & \vdots & \vdots \\ {{u_{n1}}}&{{u_{n2}}}& \ldots &{{u_{nn}}} \end{array}} \right]$$

2. Compute the priority ranking by calculating the eigenvector w corresponding to the maximum eigenvalue λ_max_, based on the judgment matrix, as given by the following equation:

$${P_w} = {\lambda _{max}} \cdot w$$

The obtained eigenvector w, after normalization, represents the priority ranking of each evaluation factor, which is also the allocation of weights.

3. To assess the reasonableness of the obtained weight allocation, a consistency check is conducted on the judgment matrix using the following formula:

$$CR=\frac{CI}{RI}$$

In the formula, CR represents the random consistency ratio of the judgment matrix, and CI represents the consistency index of the judgment matrix. The equation for calculating CR is as follows:

$$CI=\frac{{\lambda }_{max}-n}{n-1}$$

The average random consistency index (RI) of the judgment matrix is used to assess the consistency of the judgments. The Table 1 below shows the RI values for judgment matrices of orders 1 to 9 [28].

**Table 1:** The RI values for judgment matrices

*n*	1	2	3	4	5	6	7	8	9
RI	0	0	0.52	0.89	1.12	1.26	1.36	1.41	1.46

When the consistency ratio (CR) of the judgment matrix P is less than 0.1 or when λ_max_ = n and CI = 0, it is considered that P exhibits satisfactory consistency. Otherwise, adjustments to the elements in P are necessary to achieve satisfactory consistency.

The Analytic Hierarchy Process (AHP) involves conducting a total ranking to determine the combined weights of elements at a specific level in the hierarchy and their mutual influences on upper-level elements. This process utilizes the results of individual rankings at that level and calculates the combined weights of the elements. It is known as hierarchical total ranking.

In the hierarchical total ranking step, it is essential to conduct rankings layer by layer, starting from the top and moving towards the bottom. This process ultimately determines the priority order of decision alternatives by calculating the relative weights of the lowest-level elements. The hierarchical total ranking procedure is based on individual rankings within the Analytic Hierarchy Process (AHP) and follows a similar process.

$$CR=\frac{{wi}_{1}{CI}_{1}+{wi}_{2}{CI}_{2}+\varLambda +{wi}_{m}{CI}_{m}}{{wi}_{1}{RI}_{1}+{wi}_{2}{RI}_{2}+\varLambda +{wi}_{m}{RI}_{m}}$$

If the consistency ratio (CR) for the overall ranking is less than 0.1, it indicates that the consistency check for the overall ranking has passed.

### Sample relevance and basis for calculation

During the testing process, we ensured appropriate hardware conditions to ensure the accuracy and comparability of the experiments. Each subject was tested in a separate room to ensure the isolation and privacy of the testing environment. In the field of orthodontics, it is important to have a sufficiently large sample size to ensure statistically significant study results, considering the variability that may exist. To determine the sample size for this study, we used G-Power software (University of Düsseldorf, Düsseldorf, Germany). Based on the results of the pre-experiment and previous literature reports, the estimated effect size is 0.5, the test level of the experiment is set to α = 0.05, and the test power is 1-β = 0.85 [29]. The results indicate that a minimum sample size of 30 is required for this study. We included 30 subjects in the evaluation of orthodontic treatment methods, consisting of 15 patients and 15 experts. All subjects provided informed consent and their ages ranged from 20 to 47 years old. The gender distribution was equal, with an equal number of men and women.

The inclusion criteria for subjects were as follows: (1) aged between 20 and 47 years old; (2) patients with orthodontic treatment needs; (3) oral orthodontic experts with more than 5 years of clinical experience; (4) voluntarily participated in this study and signed the informed consent form.

The exclusion criteria were: (1) subjects with severe systemic diseases or mental disorders; (2) subjects who were unable to cooperate to complete the questionnaire survey; (3) patients who had received orthodontic treatment within the past year.

The patient cases were screened and selected from the hospital’s database, and the expert participants were recruited through posted notices. This study was approved by the hospital’s ethics committee, and all subjects signed informed consent forms.

The subjects rated different factors in the orthodontic treatment approach using a Likert scale [30] ranging from 1 to 9, where higher values indicate a greater impact.

## Result

**Fig. 1:**
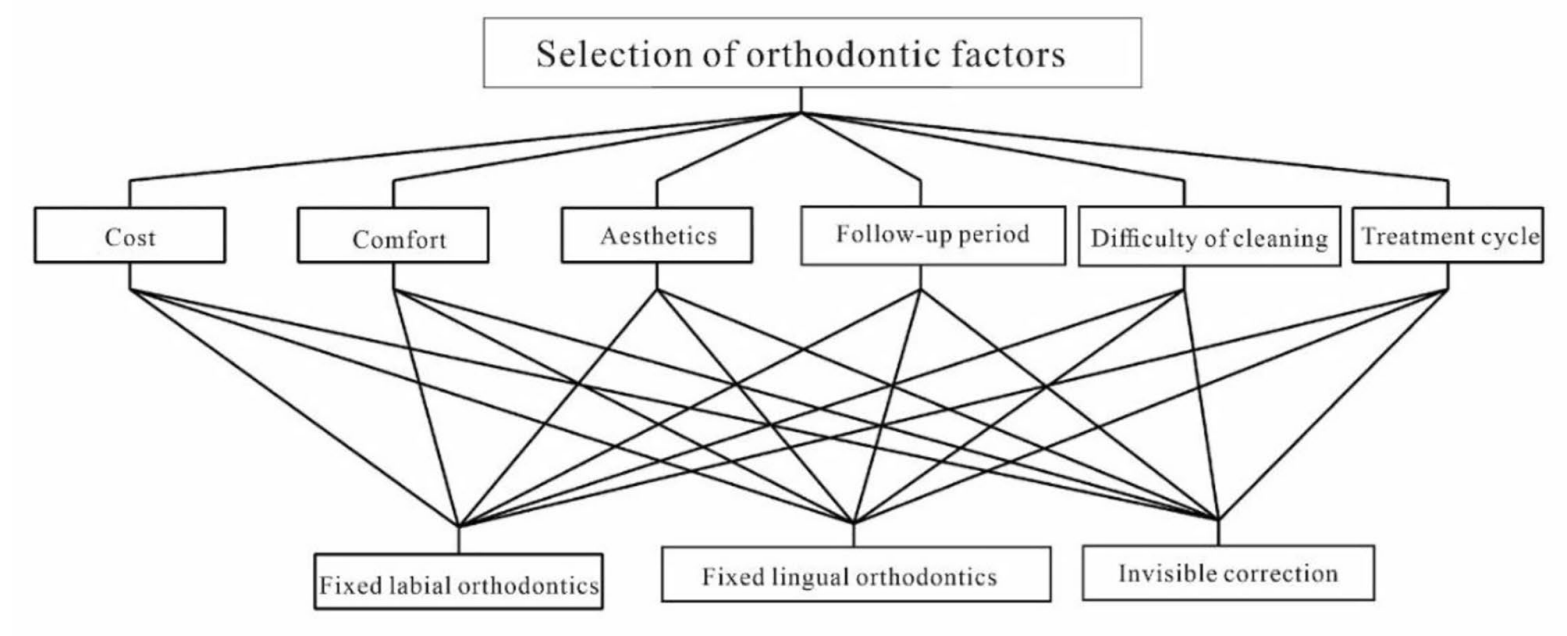
Hierarchy of orthodontic factors

The study employed a hierarchical structure to analyze orthodontic treatment methods. The hierarchy consisted of two main levels (Fig. 1). The first level, referred to as the target layer, determined the overall goal of the research and categorized orthodontic treatment methods into three underlying factors: Fixed labial orthodontics, Fixed lingual orthodontics, and Invisible correction. Moving to the criterion level, five oral clinical experts with 10 years of orthodontic experience conducted semantic analysis and identified six key characteristics of orthodontic treatment methods: cost, comfort, aesthetics, follow-up period, difficulty of cleaning, and treatment cycle. These characteristics served as the basis for subsequent application of AHP methods. The establishment of this hierarchical structure allowed the researcher to clarify the research objectives and factors, providing a clear framework for hierarchical analysis.

The optimal orthodontic plan is determined by calculating the weight of each indicator. The comprehensive scoring reveals that invisible correction has a score of 0.3923(Table 2), making it a popular treatment plan that has rapidly developed in recent times. This is followed by fixed labial correction with a weight value of 0.3312, and finally fixed lingual correction. In the middle Analytic Hierarchy Process (AHP), the factors considered include (Table 3) the cost (0.1452), comfort (0.1847), aesthetics (0.1701), follow-up period (0.1397), difficulty of cleaning (0.1843), and treatment cycle (0.1761). It is evident that comfort and cleaning difficulty are the two factors that people prioritize when undergoing orthodontic treatment, as they have relatively high scores. On the other hand, the follow-up period (0.1397) and cost (0.1452) have relatively low scores and therefore have less impact on the decision-making process for correction. And in the consistency calculation, its CR value is 0 through the consistency calculation.

**Table 2:** Conclusion table

The underlying element	Conclusion value (weight)
Fixed labial orthodontics	0.3312
Fixed lingual orthodontics	0.2765
Invisible correction	0.3923

**Table 3:** Intermediate Layer Weight Table in Group Decision-making

Node	Global weights	Sibling weights
Cost	0.1452	0.1452
Comfort	0.1847	0.1847
Aesthetics	0.1701	0.1701
Follow-up period	0.1397	0.1397
Difficulty of cleaning	0.1843	0.1843
Treatment cycle	0.1761	0.1761

## Discussion

### The influence of gender on decision-making

**Fig. 2:**
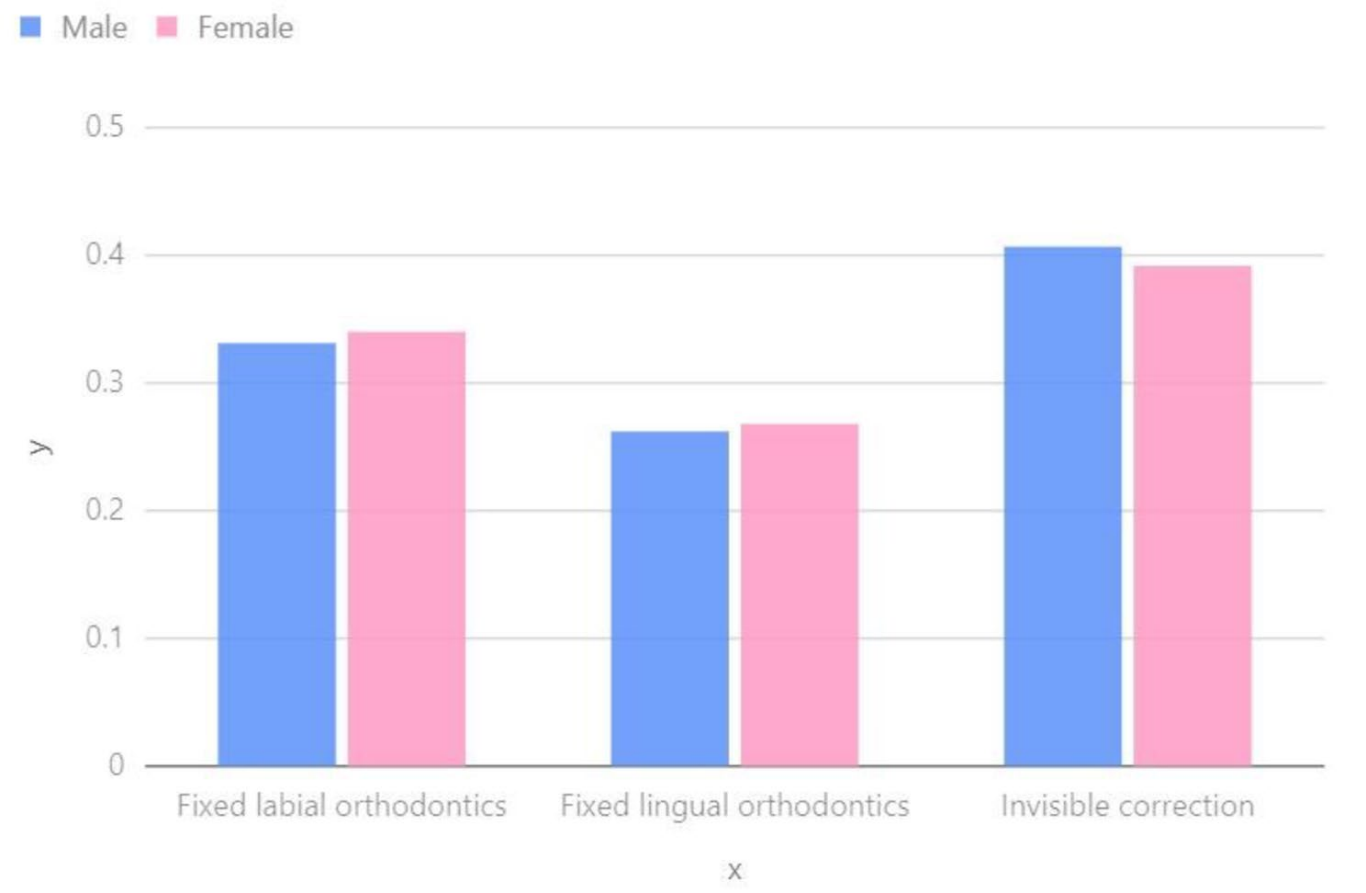
Weightage chart of gender on orthodontic treatment option selection

The study of the impact of gender on decision-making in orthodontic treatment is an important research area. The Analytic Hierarchy Process (AHP) method is used to help decision-makers balance and select the best solution among multiple factors. Gender is a potential factor that may influence orthodontic treatment decisions, particularly when considering the diverse needs and preferences of patients. As depicted in Fig. 2, both males and females tend to prefer invisible correction.

**Fig. 3:**
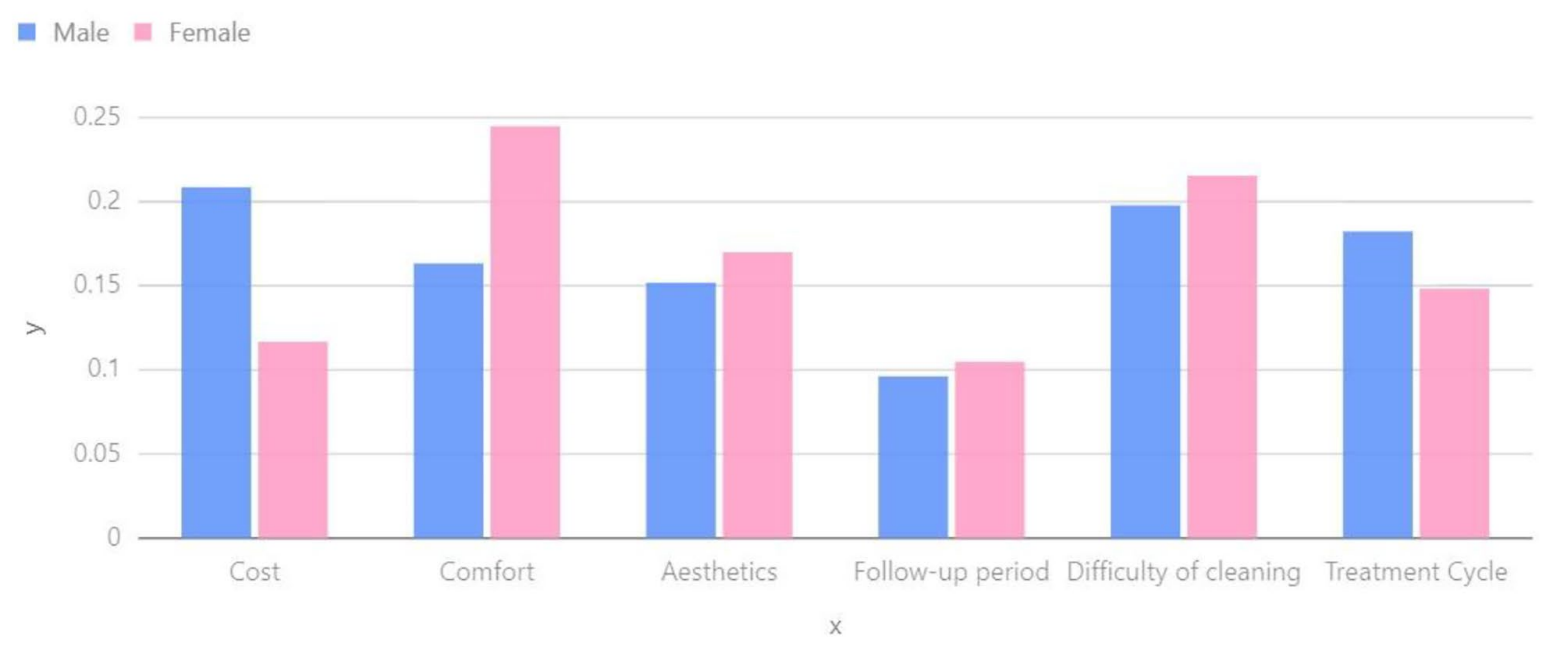
Weightage chart of gender influencing factors

Several scholars have highlighted the influence of different dental conditions on the decision to undergo orthodontic treatment and the amount individuals are willing to pay [31]. Previous studies have also suggested that women tend to prioritize their appearance more than men when it comes to orthodontic treatment, with this preference often developing in childhood [32, 33]. Furthermore, women generally have higher expectations for aesthetic outcomes during orthodontic treatment compared to men [34]. In our study, we conducted a quantitative analysis to examine gender differences, and Fig. 3 provides insights into various factors such as cost, comfort, aesthetics, follow-up period, cleaning difficulty, and treatment duration. Cost: Gender may contribute to variations in patients’ sensitivity to costs. Generally, men may exhibit a greater tendency to consider both costs and treatment outcomes.

Comfort: Gender differences may contribute to variations in treatment comfort. Research has shown that females tend to have higher sensitivity to pain and discomfort, which may impact their preferences for treatment options. Therefore, it is crucial to prioritize the needs of female patients when considering treatment comfort.

Aesthetics: Gender differences can also result in variations in perceptions of aesthetics. Females generally prioritize appearance and the aesthetics of their teeth. As a result, when considering aesthetic factors, females may prefer treatment plans that have a minimal impact on their appearance.

Follow-up period: Gender differences may influence the acceptability of follow-up appointment intervals for patients. Studies indicate that males may be more likely to prefer shorter follow-up intervals, whereas females may be more inclined to accept longer intervals. This factor could potentially impact the selection of a treatment plan.

Difficulty of cleaning: Gender differences may influence the perception of difficulty in maintaining oral hygiene. Several studies suggest that females tend to prioritize oral hygiene and cleanliness more than males. As a result, females may prefer treatment plans that have a minimal impact on oral hygiene difficulty when making decisions.

Treatment cycle: Gender differences may also influence expectations regarding treatment duration. In general, males may have a preference for shorter treatment durations, while females may be more inclined to accept longer treatment durations in order to achieve better treatment outcomes. As a result, it is important to consider gender differences when determining the appropriate treatment duration.

### Differences between doctors and patients in decision-making

**Fig. 4:**
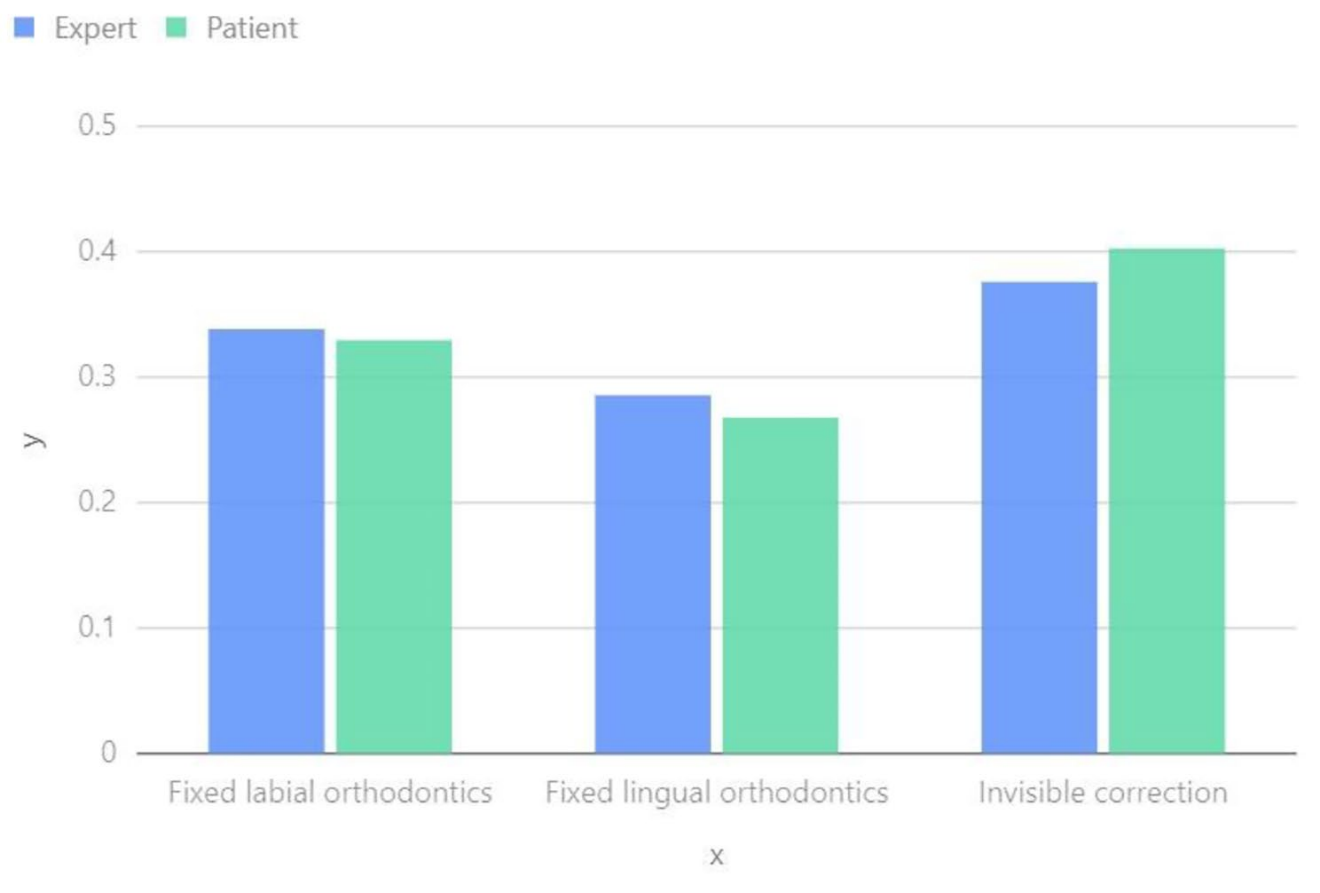
Comparison of treatment plans between experts and patients

In the three bottom-level decisions of Fixed labial orthodontics, Fixed lingual orthodontics, and Invisible correction, the differences between doctors and patients are not significant. According to Fig. 4, the order of preference is as follows: Invisible correction > Fixed labial orthodontics > Fixed lingual orthodontics.

**Fig. 5:**
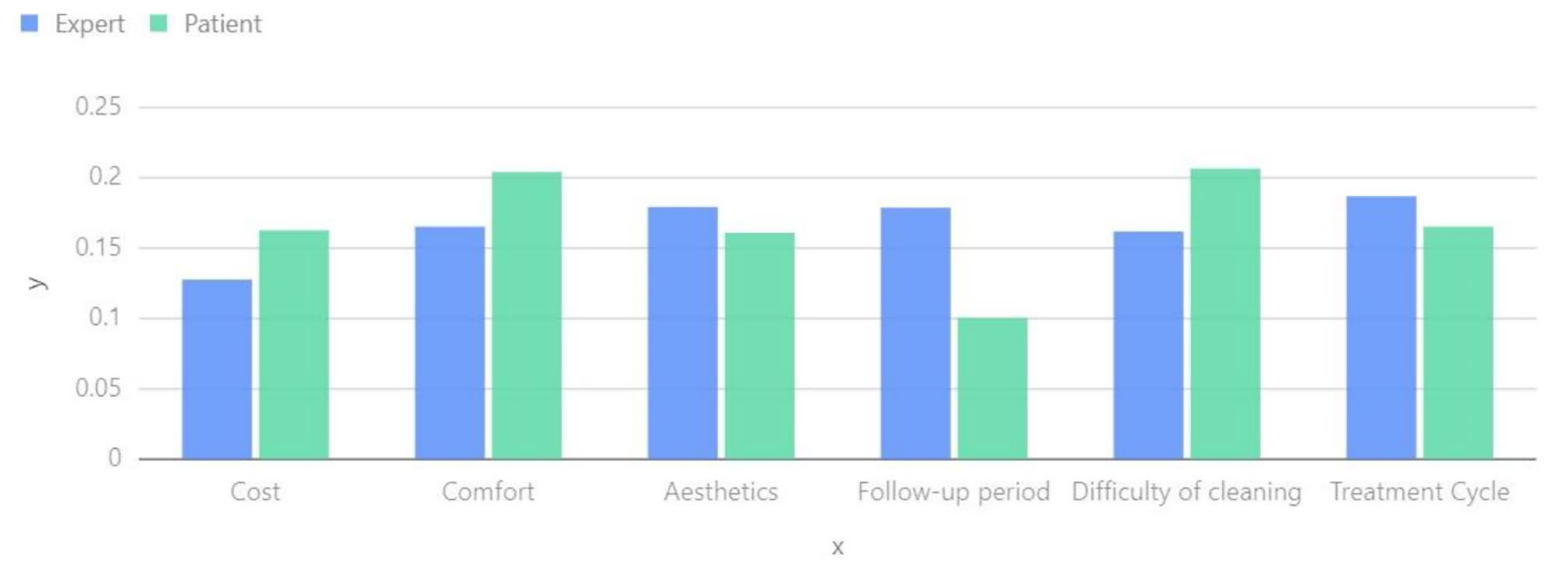
Comparison between Experts and Patients on Influencing Factors

Patients and general dentists have significantly worse initial perceptions of the aesthetics and function of their teeth compared to orthodontists [35, 36]. The personality traits of the doctor can have an impact on the patient’s orthodontic plan [37]. Effective communication between the doctor and the patient is crucial in determining the orthodontic plan during the early stages [38]. In our research, we have gained a better understanding of the patient and doctor, quantitatively analyzing the differences between them. Based on factors such as cost, comfort, aesthetics, follow-up period, cleaning difficulty, and treatment period, we have drawn the following conclusions. Please refer to Fig. 5.

Cost: Patients often prioritize treatment costs and may consider more cost-effective options, even if it means a longer treatment duration. In contrast, doctors tend to prioritize achieving the best treatment outcomes and may recommend more comprehensive treatment options, with less consideration for costs.

Comfort: Patients prioritize comfort, often preferring treatment options that minimize discomfort. Conversely, doctors primarily focus on treatment effectiveness and speed, and may consequently recommend faster but less comfortable methods.

Aesthetics: Patients frequently prioritize aesthetics, particularly when it comes to correcting visible teeth, and may have a greater tendency to opt for treatments that provide a more pleasing appearance. Doctors, on the other hand, strive to strike a balance between treatment effectiveness and aesthetics, although they may place a stronger emphasis on functionality and oral health. The importance given to aesthetics by doctors is generally less variable compared to other factors.

Follow-up period: Patients may express a preference for longer follow-up periods in order to minimize the need for frequent medical visits. Conversely, doctors may advise shorter follow-up periods in order to closely monitor the progress of treatment. This discrepancy in preference presents a notable divergence between patients and doctors.

Difficulty of cleaning: Patients may prioritize oral cleanliness and maintenance, potentially opting for a treatment approach that is easier to clean. Doctors consider the impact of treatment devices on oral hygiene but may place greater emphasis on treatment effectiveness.

Treatment cycle: Patients generally prefer shorter treatment durations in order to see improvements quickly. However, doctors recommend treatment durations based on the specific condition and complexity of the treatment plan. In some cases, they may suggest longer treatment times to ensure the best possible results.

These differences highlight the significance of maintaining a balance and fostering effective communication between doctors and patients when deciding on treatment plans. The best course of action is often achieved by finding a middle ground between the doctor’s expert recommendations and the patient’s individual needs and preferences. Consequently, shared decision-making and open communication play a vital role in orthodontic treatment.

## Conclusion

The main objective of this study is to develop an evaluation model for orthodontic treatment plans using the Analytic Hierarchy Process (AHP) and validate its feasibility and effectiveness in actual clinical practice. By establishing an AHP model based on patient needs and preferences, we provide a comprehensive and quantitative approach to facilitate the selection of optimal orthodontic treatment plans. Firstly, a thorough analysis of the patient’s oral condition and treatment requirements is conducted, and key indicators are identified using semantic analysis methods. Subsequently, an AHP model is established based on the patient’s needs, an evaluation matrix is constructed, and quantitative scoring is performed. The weight of each indicator is then calculated through mathematical calculations, and the optimal orthodontic plan is determined based on comprehensive scoring. The results demonstrate the feasibility and effectiveness of applying the AHP method in orthodontic treatment planning, offering a scientific and patient-centered decision-making tool for clinicians. The final results indicate a higher preference for invisible braces, with a relatively high comfort score among the middle-level influencing factors. This finding highlights the importance of considering patient preferences and comfort when selecting orthodontic treatment options. Therefore, special attention should be given to comfort during the treatment process. Gender should also be considered when selecting orthodontic treatment options. Studies have shown significant variations in comfort and cost, so these differences should be taken into account when recommending treatment options.

In conclusion, this research demonstrates the value of the AHP method in enhancing the objectivity and patient-centeredness of orthodontic treatment planning. By integrating multiple factors and individual needs, it provides a practical tool for clinicians to make informed decisions and improve treatment outcomes. The findings contribute to the advancement of orthodontic treatment and have implications for promoting oral health and aesthetics in clinical practice.

Therefore, Based on the research findings, it is recommended that clinicians and decision-makers in the field of orthodontics utilize the AHP method to optimize treatment plan selection, considering individual needs and integrating multiple factors. This research is expected to contribute to advancements in orthodontic treatment, improving treatment outcomes and patient satisfaction, and making practical contributions to oral health and aesthetics.

## Data Availability

All data generated or analyzed during this study are included in this published article.

## References

[1] Littlewood SJ, Mitchell L. An introduction to orthodontics. Oxford University Press; 2019.

[2] Samsonyanová L, Broukal Z. A systematic review of individual motivational factors in orthodontic treatment: facial attractiveness as the main motivational factor in orthodontic treatment. Int J Dent. 2014;2014. 10.1155/2014/938274 PMC4055094 24963296

[3] Adnan Y (2014). Positve effects for patients seeking orthodontic treatment. Int J Dent Med res.

[4] Sivakumar P (2020). Research. Advancements in technology in the field of Orthodontics. J Pharm Sci.

[5] Pithon MM, Baião FCS, Sant´ A, Paranhos LIDA, Cople Maia LR (2019). Assessment of the effectiveness of invisible aligners compared with conventional appliance in aesthetic and functional orthodontic treatment: a systematic review. J Invest Clin Dentistry.

[6] Ackerman JL, Nguyen T, Proffit WR. LW G. The decision-making process in orthodontics. Curr Principles Techniques. 2011:3–58.

[7] Goddard C (2011). Semantic analysis: a practical introduction.

[8] Klein R, Kyrilov A, Tokman M, editors. Automated assessment of short free-text responses in computer science using latent semantic analysis. Proceedings of the 16th annual joint conference on Innovation and technology in computer science education; 2011.

[9] Turney PD, Pantel P (2010). From frequency to meaning: Vector space models of semantics. J Artif Intell Res.

[10] Punyakanok V, Roth D, Yih W-t (2008). The importance of syntactic parsing and inference in semantic role labeling. Comput Linguistics.

[11] Mohammad SM. Sentiment analysis: detecting valence, emotions, and other affectual states from text. Emotion measurement. Elsevier; 2016. pp. 201–37.

[12] Hillerman T, Souza JCF, Reis ACB, Carvalho RN (2017). Applying clustering and AHP methods for evaluating suspect healthcare claims. J Comput Sci.

[13] Lee S. Determination of Priority weights under Multiattribute decision-making situations: AHP versus fuzzy AHP. J Constr Eng Manag. 2015;141(2). 10.1061/(asce)co.1943-7862.0000897.

[14] Marza B, Bratu R, Serbu R, Stan S, Oprean-Stan C (2021). Applying ahp and fuzzy Ahp Management methods to assess the level of financial and digital inclusion. Economic Comput Economic Cybernetics Stud Res.

[15] Chan HK, Wang X (2013). Fuzzy hierarchical model for risk assessment.

[16] Marttunen M, Lienert J, Belton V (2017). Structuring problems for Multi-criteria Decision Analysis in practice: a literature review of method combinations. Eur J Oper Res.

[17] Odu G (2019). Weighting methods for multi-criteria decision making technique. J Appl Sci Environ Manage.

[18] Munier N, Hontoria E. Uses and limitations of the AHP method. Springer; 2021.

[19] Sipahi S, Timor M (2010). The analytic hierarchy process and analytic network process: an overview of applications. Manag Decis.

[20] Dean M. Multi-criteria analysis. Advances in Transport Policy and Planning. Elsevier; 2020. pp. 165–224.

[21] Deif MA, Solyman AAA, Alsharif MH, Uthansakul P. Automated Triage System for Intensive Care Admissions during the COVID-19 pandemic using hybrid XGBoost-AHP Approach. Sensors. 2021;21(19). 10.3390/s21196379. 10.3390/s21196379 PMC8512533 34640700

[22] Hsieh MC, Wang EMY, Lee WC, Li LW, Hsieh CY, Tsai W (2018). Application of HFACS, fuzzy TOPSIS, and AHP for identifying important human error factors in emergency departments in Taiwan. Int J Ind Ergon.

[23] Lin CY, Shih FC, Ho YH. Applying the balanced scorecard to Build Service Performance measurements of Medical Institutions: an AHP-DEMATEL Approach. Int J Environ Res Public Health. 2023;20(2). 10.3390/ijerph20021022. 10.3390/ijerph20021022 PMC9859192 36673778

[24] Najafinasab M, Agheli L, Sadeghi H, Dizaji SF (2020). Identifying and Prioritizing Strategies for Developing Medical Tourism in the Social Security Organization of Iran: a SWOT-AHP hybrid Approach. Iran J Public Health.

[25] Pecchia L, Martin JL, Ragozzino A, Vanzanella C, Scognamiglio A, Mirarchi L, et al. User needs elicitation via analytic hierarchy process (AHP). A case study on a computed tomography (CT) scanner. BMC Med Inf Decis Mak. 2013;13. 10.1186/1472-6947-13-2. 10.1186/1472-6947-13-2 PMC3545827 23289426

[26] Yuen KKF (2014). The primitive cognitive network process in healthcare and medical decision making: comparisons with the Analytic Hierarchy process. Appl Soft Comput.

[27] Morris K (2013). Revising the declaration of Helsinki. Lancet.

[28] Bascetin A (2007). A decision support system using analytical hierarchy process (AHP) for the optimal environmental reclamation of an open-pit mine. Environ Geol.

[29] McLeod C, Fields HW, Hechter F, Wiltshire W, Rody W, Christensen J (2011). Esthetics and smile characteristics evaluated by laypersons. Angle Orthod.

[30] Jebb AT, Ng V, Tay L (2021). A review of key Likert scale development advances: 1995–2019. Front Psychol.

[31] Smith ASA, Cunningham SJ. Which factors influence willingness-to-pay for orthognathic treatment? Eur J Orthod. 2004;26(5):499–506. 10.1093/ejo/26.5.499. 10.1093/ejo/26.5.499 15536838

[32] Deli R, Macrì LA, Radico P, Pantanali F, Grieco DL, Gualano MR (2012). Orthodontic treatment attitude versus orthodontic treatment need: differences by gender, age, socioeconomical status and geographical context. Community Dentistry Oral Epidemiol.

[33] Christopherson EA, Briskie D, Inglehart MR (2009). Objective, subjective, and Self-Assessment of Preadolescent Orthodontic Treatment Need–A function of age, gender, and Ethnic/Racial background?. J Public Health Dent.

[34] Lagorsse A, Gebeile-Chauty S (2018). Does gender make a difference in orthodontics? A literature review. L’Orthodontie Francaise.

[35] McKeta N, Rinchuse DJ, Close JM (2012). Practitioner and patient perceptions of orthodontic treatment: is the patient always right?. J Esthetic Restor Dentistry.

[36] Albino J, Tedesco L, Conny D (1984). Patient perceptions of dental-facial esthetics: Shared concerns in orthodontics and prosthodontics. J Prosthet Dent.

[37] Bedair TM, Thompson S, Gupta C, Beck FM, Firestone AR (2010). Orthodontists’ opinions of factors affecting patients’ choice of orthodontic practices. Am J Orthod Dentofac Orthop.

[38] Kawanichi LY, Suga U, Kruly PC, Fujimaki M, Provenzano M, Terada RSS (2017). Patient satisfaction after orthodontic treatment: a systematic review. Brazilian Dent Sci.

